# Armadillo 1.1: An Original Workflow Platform for Designing and Conducting Phylogenetic Analysis and Simulations

**DOI:** 10.1371/journal.pone.0029903

**Published:** 2012-01-11

**Authors:** Etienne Lord, Mickael Leclercq, Alix Boc, Abdoulaye Baniré Diallo, Vladimir Makarenkov

**Affiliations:** 1 Département d'informatique, Université du Québec à Montréal, Montréal, Canada; 2 Département de sciences biologiques, Université de Montréal, Montréal, Canada; Montreal Botanical Garden, Canada

## Abstract

In this paper we introduce Armadillo v1.1, a novel workflow platform dedicated to designing and conducting phylogenetic studies, including comprehensive simulations. A number of important phylogenetic and general bioinformatics tools have been included in the first software release. As Armadillo is an open-source project, it allows scientists to develop their own modules as well as to integrate existing computer applications. Using our workflow platform, different complex phylogenetic tasks can be modeled and presented in a single workflow without any prior knowledge of programming techniques. The first version of Armadillo was successfully used by professors of bioinformatics at Université du Quebec à Montreal during graduate computational biology courses taught in 2010–11. The program and its source code are freely available at: <http://www.bioinfo.uqam.ca/armadillo>.

## Introduction

Bioinformatics is a fast-evolving field that encompasses molecular biology, biochemistry, computer science, mathematics and statistics [1]. It has emerged as a field of its own due to the dramatic increase and complexity of available genomic data [2]. Phylogenetics, which is a subfield of bioinformatics and molecular biology, studies evolutionary relationships between organisms based on their molecular or morphological proximity and presents those relationships through illustrations called phylogenetic trees (or phylogenies) [3].

The development of a variety of bioinformatics and phylogenetic algorithms has led to the design of numerous computer applications often generating different results when solving the same computational biology problem [2], [4]. Thus, modeling and performing computational biology simulations can be a very challenging task due to the quantity and diversity of available tools and genomic databanks. Tutorials and examples have been commonly distributed with phylogenetic and bioinformatics applications, whereas online training courses can be found in different web directories, such as Bioinformatics Link Directory [5] for example. However, “standard practices” for bioinformatics and phylogenetic analysis have not been strictly defined (apart from a number of specific fields [6], [7]); each analytical step can be carried out using a variety of methods and tools [2]. Moreover, when conducting their experiments and simulations, computational biologists have to cope with programs' limitations and data integration issues [1]. In addition, incorrect outcomes of biological data analysis can arise when accessible, but wrong, tools and models are used [8].

**Figure 1:**
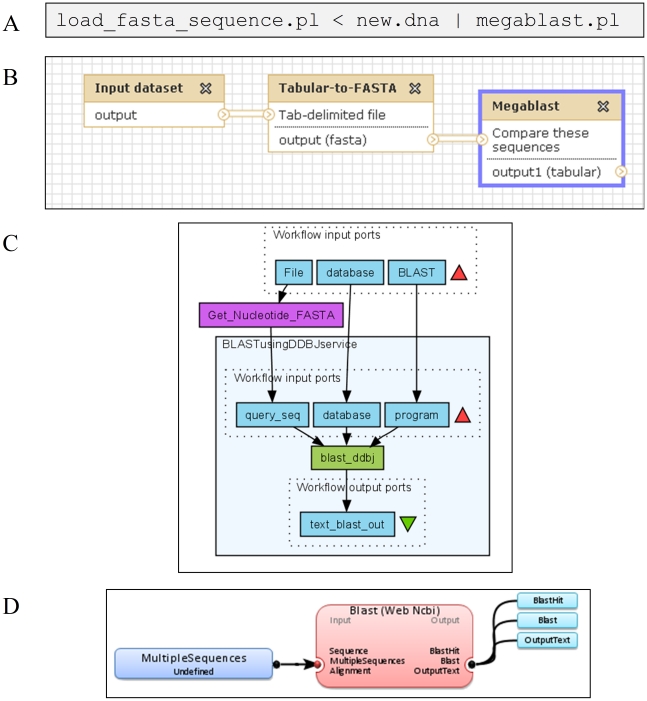
Comparison of four different bioinformatics platforms for sequence search (i.e., the BLAST algorithm is used in all of them). Panel (A) presents a standard pipeline using Perl scripting; Panels (B, C, D) show different workflow designs for the sequence search operation provided by Galaxy (B) [10], Taverna (C) [27] and the introduced Armadillo workflow platform (D).

Meanwhile, the increasing use of genomic and phylogenetic data fuels the need for pipeline managing software. For instance, Cicarelli et al. [9] developed an automatable procedure for reconstructing the tree of life with branch lengths comparable across the three species domains which can be represented as a typical bioinformatics workflow encompassing the following major tasks (also see Fig. 1. in [9]): Selection and preparation of marker gene families, generation of concatenated multiple sequence alignments (supermatrix approach), systematic detection and removal of horizontal gene transfers, phylogenetic tree reconstruction and evaluation. Philippe et al. [10] warn, however, that automated phylogenetic analysis has its own pitfalls and caveats. Thus, all automatically generated data must be “manually” verified and corrected, if necessary, before proceeding to their further treatment or interpretation.

A typical bioinformatics task can be described as a workflow pipeline in which resources or data are sequentially processed by a series of dedicated tools [1]. A workflow pipeline usually includes: (1) Data acquisition, (2) data analysis and (3) report and results generation steps [4]. Figure 1 presents an example of a standard task carried out when searching for DNA sequences similar to a given one. Such a task requires a combination of three steps: (1) Input of the given DNA sequences in a particular data format, (2) its conversion to a compatible format according to the tools being used, and finally, (3) execution of an appropriate online or local database query using a sequence matching algorithm such as BLAST [11]. Hence, building solid bioinformatics studies requires the integration of several computational tools, which can include public or commercial computer applications. The following major issues can arise while integrating them into a single pipeline: Communication protocols between applications (e.g., file formats and command line options), computational requirements of embedded algorithms, limited or inexistent electronic access to public or private biological databanks, software running under different operating systems and non-standardized result reports generated by the applications [4]. Life scientists with no prior knowledge in computer science are either limited to the basic usage of existing tools or need to learn a script language, such as Perl (Fig. 1A), or a programming language, such as C or Java [12], in order to implement their experiments.

Several computational tools have been introduced to help bioinformatics researchers perform complex tasks: (1) Web-based applications, such as Galaxy [13] (Fig. 1B) or ENSEMBL-European Bioinformatics Institute platform (EBI) [14], providing users with a multi-processor cluster access and user-friendly interface. In the same context, web-only portals such as Phylogeny.fr [15], Phylemon [16] and Bioextract.org [17] allows the creation of simple execution pipelines intended for phylogenetic inference (e.g., loops and conditional operations are not allowed in those pipelines), along with the AIR [18] web server, which is intended for conducting phylogenomics analysis using supermatrix methodology; (2) Dedicated programming libraries, BioPerl [19], BioJava [20], etc., written in popular script languages in order to facilitate programming tasks; (3) Multi-task applications (i.e., applications merging different programs) or computer packages including a variety of general bioinformatics algorithms, such as MEGA [21], Geneious [22] and Mesquite [23]. The examples of bioinformatics tasks that can be performed using the three latter packages include multiple sequence alignment, phylogenetic inference, short-read assembly, recombination analysis, reconstruction of ancestral states, simulation of character evolution and detection of deep coalescence. In the same way, the packages PAML [6], PAUP [7], PHYLIP [24] and T-Rex [25] are specialized multifunction software focusing on phylogenetic analysis.

While the first and third types of tools rely on the manipulation of diverse data formats and manual execution of different applications to answer real-life biological questions, the second type of tools requires deep knowledge of programming languages. An emerging approach in the Life Science community is the development of workflow platforms (Fig. 1B–1D) [26] which can be used for designing and running comprehensive simulation studies as well as for educational purposes.

Workflow platforms consist of connected processes or applications. They were originally implemented and used in financial and business environments [27], [28]. Workflows have been also actively employed to chain together specialized programs in order to create dataflow for software development, including scientific applications [27]. They provide a suitable environment for defining and managing the coordination and allocation of process activities [29]. Workflow platforms expand from simple execution managers to complex systems often allowing for conditional dataflow scheduling and task distribution [1], [13]. Such platforms also simplify the automation of error-prone tasks, data collection including refactoring and organization of input/output, data processing and results visualization [27], [30]. The examples of workflow platforms dedicated to bioinformatics include the web-based platform Galaxy [13] (Fig. 1B) and desktop-based platform Taverna [27] (Fig. 1C). Both of them contain a specific workflow language and particular computational model supporting automation. While Galaxy is based on private server architecture, Taverna relies on well developed web-services.

Previous studies showed that workflow platforms can be also useful in finding solutions, answering questions and facilitating “just-in-time” learning and demonstration [31]. Several workflow platforms have been dedicated to education [29], [31]–[34]. They provide teachers with different web-based (e-learning, tele-learning) or desktop-based protocols helping them create appropriate educational content [29], [35], while keeping record of students' results, failures and attempts [35]. Most of educational workflows intend either to enhance cognition of students or verify the quality of the learning process [36]. To the best of our knowledge, no workflow platforms dedicated to bioinformatics, or phylogenetic, education have been proposed. Thus, the development of a workflow application encapsulating and managing the most popular bioinformatics algorithms and data formats would be a very important task for this vital Life Science field. Such a platform would permit students to ignore the “cooking” programming and to focus directly on actual goals of their projects. It would ideally satisfy to a WYSIWYG (What You See Is What You Get) principle, or more precisely in our case, to a WYPIWYG (What You Pipe Is What You Get) principle [2].

In this article we describe Armadillo, an original workflow platform dedicated to modeling complex phylogenetic as well as certain general bioinformatics experiments. Armadillo proceeds by a local execution of popular phylogenetic applications. It allows users to quickly design large-scale computational biology experiments, facilitating data format management, electronic transactions with biological databanks and manual conversion of inputs and outputs of several major bioinformatics applications. Our platform proposes an elegant graphical user interface (GUI), conventionally presenting the available applications as single boxes and computational pipelines as box interconnections. Interconnections represent the data acquisition events and create a flow involving different existing applications (see Fig. 1D). In the following sections we will describe how Armadillo can be used to carry out phylogenetic analysis and facilitate phylogenetic and general bioinformatics learning.

## Methods

### General description of Armadillo

**Figure 2:**
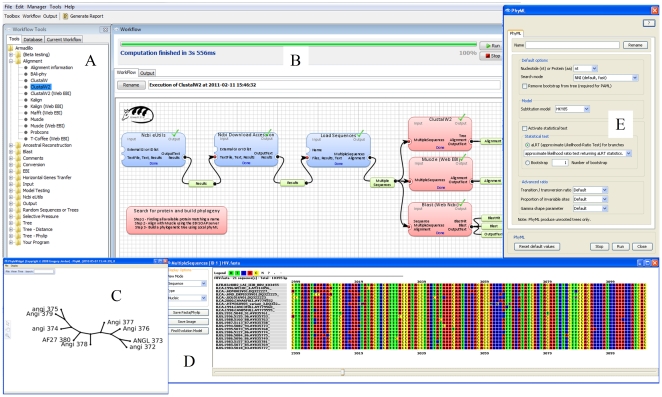
Overview of the graphical user interface of the Armadillo workflow platform. Panel (A) presents the available tools. All these tools can be used as drag-and-drop components in the workflow. Panel (B) presents the main view of the workflow design. Panel (C) presents a picture of a phylogenetic tree (i.e., phylogeny or evolutionary tree) displayed using the PhyloWidget application [40]. Panel (D) shows an integrated sequence viewer. Panel (E) shows an example of a custom component view allowing an easy configuration of the user pipeline.

The Armadillo workflow platform (version 1.1) has been developed using the processing library for GUI operations (Fig. 2). Similarly to the other existing bioinformatics platforms, such as Taverna [27] or Galaxy [13], the components representing either datasets or bioinformatics applications can be linked together to create a dataflow by performing drag-and-drop box-connection operations (Fig. 2A and 2B). The Armadillo platform integrates a sequence viewer and provides access to phylogenetic tree interference and manipulation applications (Fig. 2C and 2D). The configuration of each application is performed via a custom dialog box (Fig. 2E) which facilitates the use of the most commonly used features. Armadillo does not require an Internet access for most of its operations. Developed in Java, it runs under the Windows OS and Mac OS X (including Leopard and Lion) operating systems and comprises several popular Windows and Apple-Macintosh compiled applications. The minimum system requirements are stated on the home page of the Armadillo website (for more details, see: http://www.bioinfo.uqam.ca/armadillo). The installation of Armadillo can be performed in a single step which includes the set up of many popular phylogenetic programs (see Table 1). Certainly, the user has to cope with particular memory and parameters limitations imposed by the applications included in the Armadillo platform as well as with the RAM overflow that can be caused by executing those applications on large datasets. By using the *Preference→Advanced* option, the user can specify the maximum amount of RAM memory that can be used by Java.

**Table 1:** Bioinformatics applications and services included in Armadillo v1.1.

Bioinformatics tasks	Applications and services
**Online Databank Access**	
National Center for Biotechnology Information (NCBI)	Access to database search and data downloads through the EUtils Web-services^b^
ENSEMBL-European Bioinformatics Institute (EBI)	Access to database search through EBI-Eye [58] and data downloads through dbFetch [14]
HUGO Gene Nomenclature Committee	Access to database search and downloads of human genes information
**Multiple Sequence Alignment**	BAli-phy [59], ClustalW [56], ClustalW2 [60], Kalign [61], Muscle [44], ProbCons [45]
**Detection of Horizontal Gene Transfers**	HGT Detection [51], PhyloNet – RIATA_HGT [52], LatTrans [53]
**Phylogenetic Tree Inference**	fastDNAml [62], DNAml-Erate [63], Garli [64], MrBayes [65], PhyML [46], PHYLIP package (11 applications included) [24], random tree generation program [25]
**Phylogenetic Tree Visualization**	PhyloWidget [40], Archaeopteryx [41], ScripTree [66]
**Evolutionary Model Selection**	jModelTest [55], ProtTest [57]
**Selective Pressure Analysis**	PAML v4.4 [6]
**Local Alignment and Sequence Search with BLAST**	BLAST (Local and Web at EBI and WTSI, and NCBI) [11]

See the Armadillo website for the complete list of included applications^a^.

a Up-to-date list of included applications is available at: http://adn.bioinfo.uqam.ca/armadillo/included.html.

b NCBI EUtil is available at: http://www.ncbi.nlm.nih.gov/entrez/query/static/esoap_help.html.

As highlighted by Stevens et al. [4], life cycle of *in silico* experiments consists of different key points that need to be addressed, including: Performing experimental design, carrying out executions, interpreting the obtained results and presenting or publishing related articles or research reports. The Armadillo platform has been built in order to ease many of such *in silico* tasks. Mention that all application inputs taken from application outputs are written into a single “project” storage file. Storage files are implemented by means of a Structured Query Language (SQL) compatible database, built using the Xerial Java implementation of SQLite [37]. The project file database can also be displayed on a website to allow users to share their results or compare different workflow strategies. Not supporting distributed databases can seem odd in our cloud computer epoch [38], but the main goal of this project was to ease the phylogenetic workflow design without having to create users, groups or ensure a secure access to databanks. However, we consider all these options as possible future extensions of our workflow platform. The single file system used in Armadillo also facilitates the organization of course material allowing for a quick creation of exercises, datasets and working diagrams. Text and HTML files can be directly added to any Armadillo workflow. The platform also supports annotation of individual data components, and thus permits teachers, students and researchers to comment the results of their analyses. Finally, the Armadillo architecture can be also used during an SQL language course since all SQL queries are displayed and executed directly within the platform.

### Applications included in Armadillo 1.1

The first version of the Armadillo platform supports various sequence data formats via the inclusion of the ReadSeq application developed by Gilbert [39]. For example, the Newick format is used as a default format to represent phylogenetic trees. The tree visualization can be generated using either the PhyloWidget viewer [40] or the Archaeopteryx application [41] supporting the XML, Tree-of-Life and Nexus file formats. Several popular tools for solving fundamental phylogenetic problems, such as reconstruction of species evolution from molecular sequences (i.e., phylogenetic inferring), multiple sequence alignment algorithms and database queries using BLAST constitute the core applications included in Armadillo 1.1 (see Table 1 for the complete list of available applications). For instance, the PAML [6] and PROTML (from the PHYLIP package [24]) programs provided with Armadillo can deal with different codon models. The Gblocks application [42], [43] can be used to improve the quality of multiple sequence alignments through removing divergent and ambiguously aligned blocks. Custom functions can be also added to the workflow since the runtime compilation and execution of Java programming source code are integrated in our platform. This feature is available via the *Tools→Your Program→Custom program* option. A description of the basic functions of each application included in Armadillo is accessible by clicking the application information button; this button is represented by the “?” symbol.

**Figure 3:**
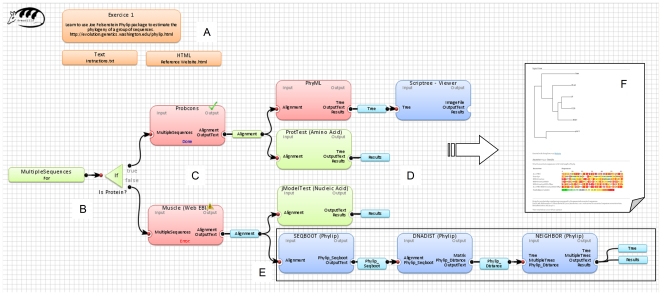
An example of a bioinformatics solution created with Armadillo. Panel (A) presents available comments and support files (available in the text and HTML formats). Panel (B) presents the beginning of the workflow and the *if* control used to select between different alternatives in the dataflow. Panel (C) shows how different multiple sequence alignment applications can be modeled. Panel (D) illustrates the use of different colors to annotate different parts of the workflow in order to facilitate the learning process. Panel (E) presents an example of a phylogenetic pipeline. Panel (F) displays an example of obtained results (i.e., results report).

The results generated by any application embedded in an Armadillo computational pipeline can be accessed and verified as well. Our platform proceeds by the validation of results of each application during the workflow execution. Once the computation is over, a complete report is generated to present the detailed descriptions of different steps as well as the corresponding results (Fig. 3). Such a report includes all obtained results, application states, error encountered (if any) and application outputs obtained at all intermediate steps of the analysis. Reports are presented through the hyperlinks to the associated HTML files. An example of a complex task used in our simulations is presented in Figure 3. Here the multiple sequence alignment applications (i.e., Muscle [44] and ProbCons [45]) provide the input sequence alignments for the phylogenetic tree reconstruction algorithms (i.e., PhyML [46] and NJ [47]), which then can be followed by several applications for validating and comparing the obtained tree topologies.

It is worth noting that scientific workflows are usually data-intensive [38]. They can take advantage of the Web 2.0 features either through datasets acquisition, or through distributed computational services. This allows users to free local workstation computational resources for further analyses. We also included to the first version of Armadillo the access to two major data source and web service providers: GenBank maintained by the National Center for Biotechnology Information (NCBI) and Ensembl maintained by European Bioinformatics Institute (EBI) and the Wellcome Trust Sanger Institute (WTSI) (see Table 1).

In Table 2, we compare the main features of the introduced Armadillo platform with those available in the other popular bioinformatics workflow software, including Ergatis [48], Galaxy [13], Kepler [49], LONI [50] and Taverna [36]. They concern workflow design, experimental data organization and possibility of adding new applications.

**Table 2:** Comparison of the main features provided by Armadillo v1.1 with those available in the Taverna [27], Galaxy [13], LONI [50], Ergatis [48] and Kepler [49] bioinformatics workflow platforms.

	Workflow design		Data management				Platform expansion	
Platform	*Drag-and-Drop*	*Loops/Conditional executions*	*Client-Server Model*	*Data organization*	*Data search function*	*Repetition of experiments*	*Open platform*	*Addition of new applications*
*Armadillo*	Yes	Yes	No	Yes	Yes	Yes	Yes	Yes^a^
*Taverna*	No	Yes	No	No	No	Yes	Yes	Yes^a^
*Galaxy*	Yes	No	Yes	Yes	Yes	Yes	No	No
*LONI*	Yes	Yes	Yes	Yes	Yes	Yes	No	Yes
*Ergatis*	Yes	Yes	Yes	No	No	Yes	Yes	Yes
*Kepler*	Yes	Yes	No	No	No	Yes	Yes	Yes

a Addition of new applications through Web Services or Java programming.

### Case study: Using Armadillo to infer phylogenetic trees

Phylogenetic tree inference is an important step of many bioinformatics projects such as those dedicated to detection of horizontal gene transfers [51]–[53]. The case study we present here highlights different steps which are necessary for building a phylogeny of the adiponectin protein using the Armadillo platform. Adiponectin, also denoted as Acrp30, apM1, GBP28 or ADIPOQ, due to its discovery by four different research groups, is a 244-amino acid protein primarily secreted by the white adipose tissue. This protein is known for its pleiotropic effects and is involved in obesity-related disorders: Type 2 diabetes mellitus, metabolic syndrome and atherosclerosis. Recently, it has been suggested that adiponectin can also be cardioprotective and has anticancer properties [54].

#### Step I: Creating an adiponectin protein dataset

**Figure 4:**
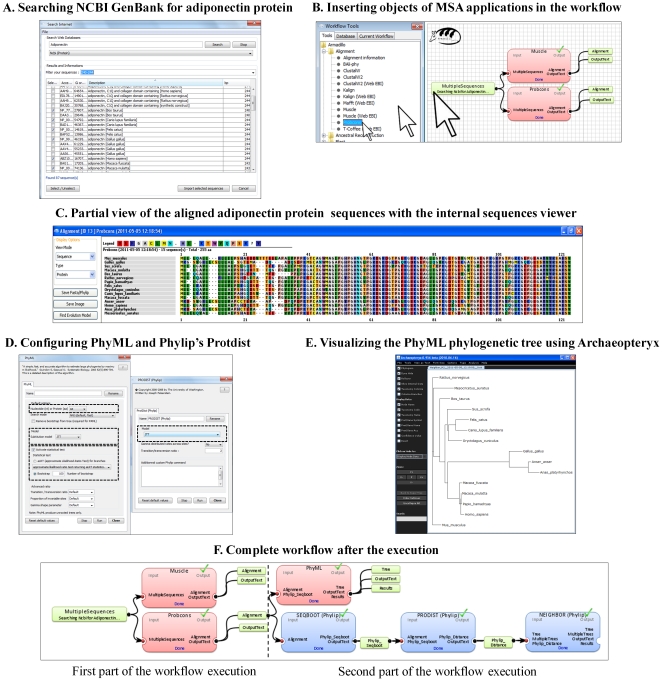
A quick view of different steps needed for phylogenetic inference with Armadillo. Step A: Search dialog box allowing for direct access to different Internet databases. Step B: Creating and interconnecting individual components by means of drag-and-drop operations. Muscle and ProbCons multiple sequence alignment applications are presented here. Step C: Representing the aligned sequences using an internal sequence viewer. Step D: Configuring the options of the PhyML and ProtDist applications prior to phylogenetic inference. Step E: Visualizing the resulting PhyML phylogenetic tree using the Archaeopteryx tree viewer. Panel (F): Displaying the complete computational workflow after a sequential execution of the first (multiple sequence alignment algorithms) and second (phylogenetic tree inference algorithms) workflow parts.

Inferring a phylogenetic tree is a three-fold process including: (1) creation of a sequence dataset, (2) alignment of the selected sequence data, and (3) inference of a phylogenetic tree from the obtained multiple sequence alignment using a relevant evolutionary model to represent evolutionary history of the considered organisms [55]. The first step here involves searching for the amino acid sequences to be aligned. Starting with a new empty project (*File*→*New project*), one can use the main application menu: *Manager*→*Sequences* to open *Sequence Manager* (Fig. 4A). In the sequence manager dialog box, the option: *File*→*Import from Internet* should be used. This will open a new dialog box named *Search Internet* which allows access to data from three major Internet sequence databanks: HUGO (Human Gene Nomenclature Committee), GenBank (supported by NCBI) and Ensembl (see Table 1). For the presentation purpose, we will choose in the database dropdown menu the NCBI Protein database option with the keyword Adiponectin. Once the database search is over, one can filter the search results by retaining for further analysis only the adiponectin sequences and eliminating the derived peptides and adiponectin receptors (i.e., AdipoR1, AdipoR2). To proceed, one has to enter in the *Filter your sequences* field the range “240–244” and order the sequences by *Description*. Following the filtering operation, the selected sequences can be downloaded from GenBank using the option *Import selected sequences* located at the bottom of the dialog box. The sequence manager can be closed afterwards.

#### Step II. Generating multiple protein sequence alignments

Generation of a correct alignment of the selected sequences is the second key step of the tree building protocol [44], [45]. To align the downloaded protein sequences, one can go to the left workflow toolbox (Fig. 4B), select the newly added *MultipleSequences object* in the *Tool*→*Database panel* and drag and drop it to the workflow creation area (WFCA). Two multiple sequence alignment applications were used in this example to treat the adiponectin protein dataset. First of them is the Muscle program [44], which usually shows a greater accuracy than the popular ClustalW algorithm [56] when aligning protein sequences, and the second one, the ProbCons program [45] based on probabilistic modeling. To use those programs, one need to incorporate them into the workflow by going into the *Toolbox*→*Tools* (Fig. 2A) panel and expanding the *Tools*→*Alignment subtree* (Fig. 4B). Once the Muscle and ProbCons applications are located, they can be dragged and dropped onto WFCA (Fig. 2B) and connected to the previously added *MultipleSequences object*. One can now execute the workflow using either the *Run* button located at the top-right corner of WFCA (Fig. 2B) or the *Execution*→*Run* option from the main application menu. Once all execution steps are completed, the progress bar at the top of WFCA will advance to the 100% mark (Fig. 2B). One can visualize the obtained results (Fig. 4C) by either expanding the program output in WFCA and then double-clicking on the resulting alignment or by going into the workflow *Toolbox*→*Current Workflow* panel, then expanding the Muscle and ProbCons programs subtree, right-clicking on the alignment of choice, and finally selecting the *Details* or *View Graphic* option in the contextual pop-up menu.

#### Step III. Inferring the adiponectin phylogenetic trees

Once the user is satisfied with one of the obtained sequence alignment, he/she can start inferring phylogenies. Here we present two alternative solutions: The first tree will be obtained using a fast a very popular maximum likelihood method implemented in the PhyML application [46], while the second tree will be generated by the Neighbor-Joining method [47] available in the PHYLIP package (i.e., the Neighbor application [24]). To perform the tree inferring, one can go to the *Toolbox*→*Tools* panel, select the *Tree*→*PhyML* option, drag and drop the application onto WFCA and then connect it to the newly created alignment. In the same way, one can go to the *Tree*→*PHYLIP* subtree, drag and drop the Seqboot, Protdist and Neighbor applications onto WFCA and thus create a second tree inferring pipeline by connecting these applications as shown in Figure 4F. To customize the program execution parameters, one can double-click on the corresponding box of WFCA. For example, in the case of the PhyML program and adiponectin dataset, one could select the following parameters in the default option area: aa (protein) - for the dataset, JTT model - for the substitution, and 100 - for the number of bootstrap replicates (see Fig. 4D). Alternatively, using a PHYLIP package application one could also perform the *Model Testing*→*ProtTest* [57] (see Fig. 3) test to identify the most appropriate evolutionary model characterizing the considered multiple sequence alignment. Once all execution steps are completed, the resulting phylogenetic trees (in the Newick format) can be viewed by double-clicking on the generated output file. On the other hand, a graphical representation of the obtained phylogenetic trees can be generated by right-clicking on the tree output file and selecting the option *View Tree* in Archaeopteryx or *View Tree* in PhyloWidget in the contextual pop-up menu (see Fig. 4E). Obviously, the whole computational pipeline as well as the obtained intermediate and final results can be saved and then easily modified to carry out further analyses.

## Results and Discussion

In this article we described Armadillo (version 1.1), an original workflow platform dedicated to designing and performing phylogenetic analysis and simulations. The new platform implements an intuitive approach for task automation and simulation design. The current version of Armadillo allows the creation of repetitive workflows, while ensuring the compatibility of numerous phylogenetic and certain general bioinformatics applications distributed with the platform. For instance, Armadillo can be used to perform multiple gene analyses (for example, by means of the *for* loop allowing for processing multiple gene files). Armadillo was first used to design and carry out a simulation study comparing the most popular methods for inferring horizontal gene transfer events (i.e., HGT-Detection [51], RIATA_HGT [52] and LatTrans [53] algorithms were compared in a 2010 Systematic Biology article [51]). The new platform was then successively employed as a support tool during the 2010–11 academic year by professors of bioinformatics at Université du Québec à Montréal in the framework of several graduate computational biology courses. Armadillo has been also recently used to perform a complex microRNA classification study conducted by the bioinformaticians of Université du Québec à Montréal as well as a HIV selective pressure study carried out by the researchers of Sainte-Justine Hospital of Montreal. Our software provides a number of interesting features unavailable in the Taverna [27] and Galaxy [13] workflow platforms (see Table 2) and includes the tools for solution sharing, genomic databank transactions and program integration. The current version of Armadillo has been released under the Open Source GNU General Public License. The source code, several tutorials and application examples are provided on the Armadillo website: http://www.bioinfo.uqam.ca/armadillo. It is worth noting that we have also implemented a Wiki-style user guide in order to enhance the software learning capacities. Such an interactive manual allows users to suggest the addition of new options and applications as well. Mention that Armadillo can be easily extended by other developers. This extension can concern either the workflow platform itself or the integrated data types and included bioinformatics tools. In the future, we plan to add to Armadillo different multimedia files to increase its learning capacity. We also envisage including in the new platform a data management application and providing users with a data storage facility. All these improvements of Armadillo will aid teachers to create specific educational toolkits for Life Science students. They will also allow Armadillo to become a tool of choice for conducting complex phylogenetic as well as broad-spectrum bioinformatics simulations for many molecular and evolutionary biologists, bioinformaticians and biostatisticians.
